# Supplementary material for: Inorganic Nitrogen Transport and Assimilation in Pea (Pisum sativum)
Source: Genes (Basel). 2022 Jan 17;13(1):158. doi: 10.3390/genes13010158 (PMC8774688; doi:10.3390/genes13010158)
Supplement: Supplementary file 1 [file genes-13-00158-s001.zip › Supplemental1_ Figures and Table.pdf]

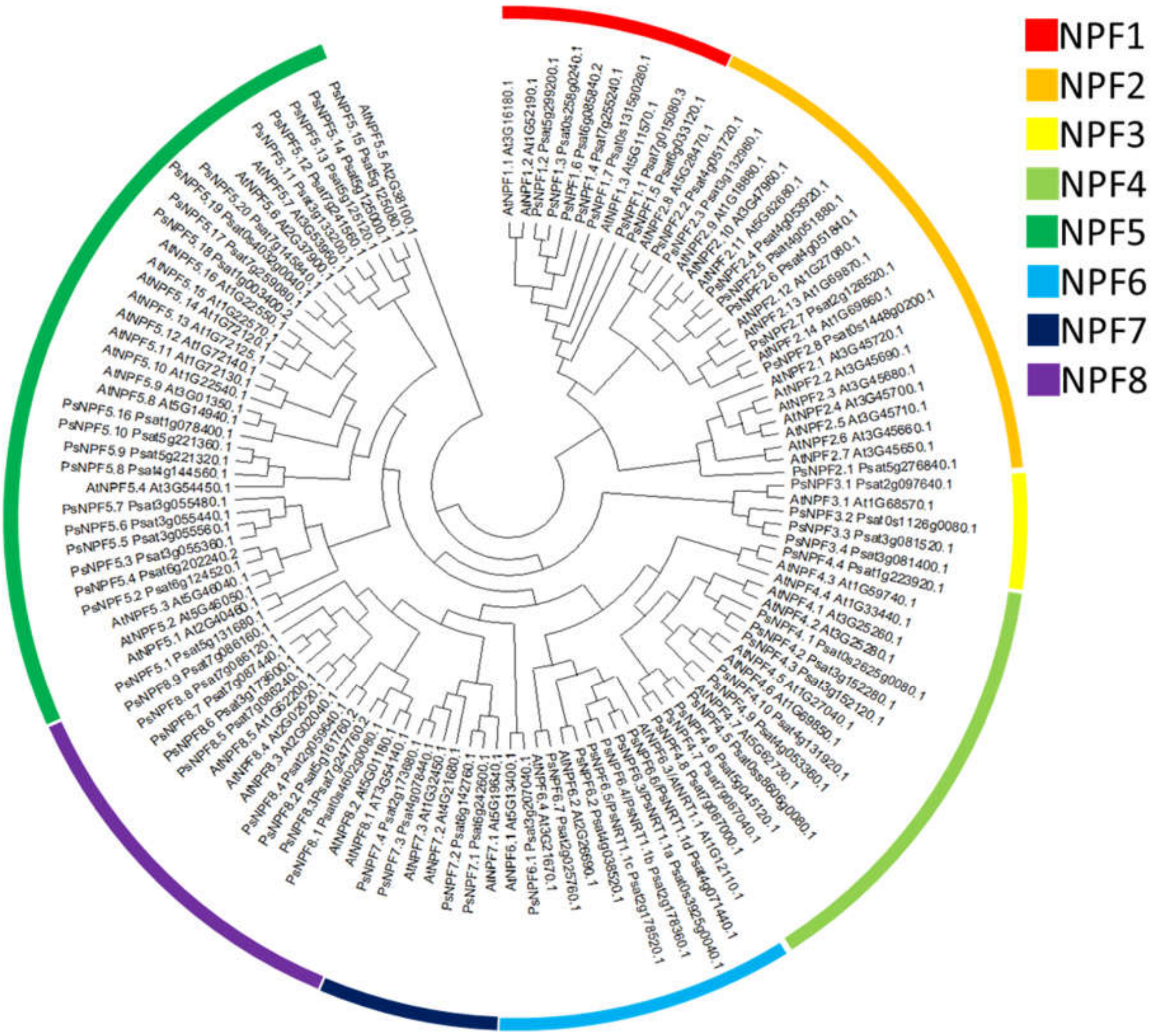

Supplemental Figure S1. A phylogenetic tree of NRT1/ PTR FAMILY (NPF) proteins from *Arabidopsis thaliana* and *Pisum sativum*

Eight NPF families are indicated by red (NPF1), orange (NPF2), yellow (NPF3), light green (NPF4), green (NPF5), blue (NPF6), indigo (NPF7) and purple (NPF8).

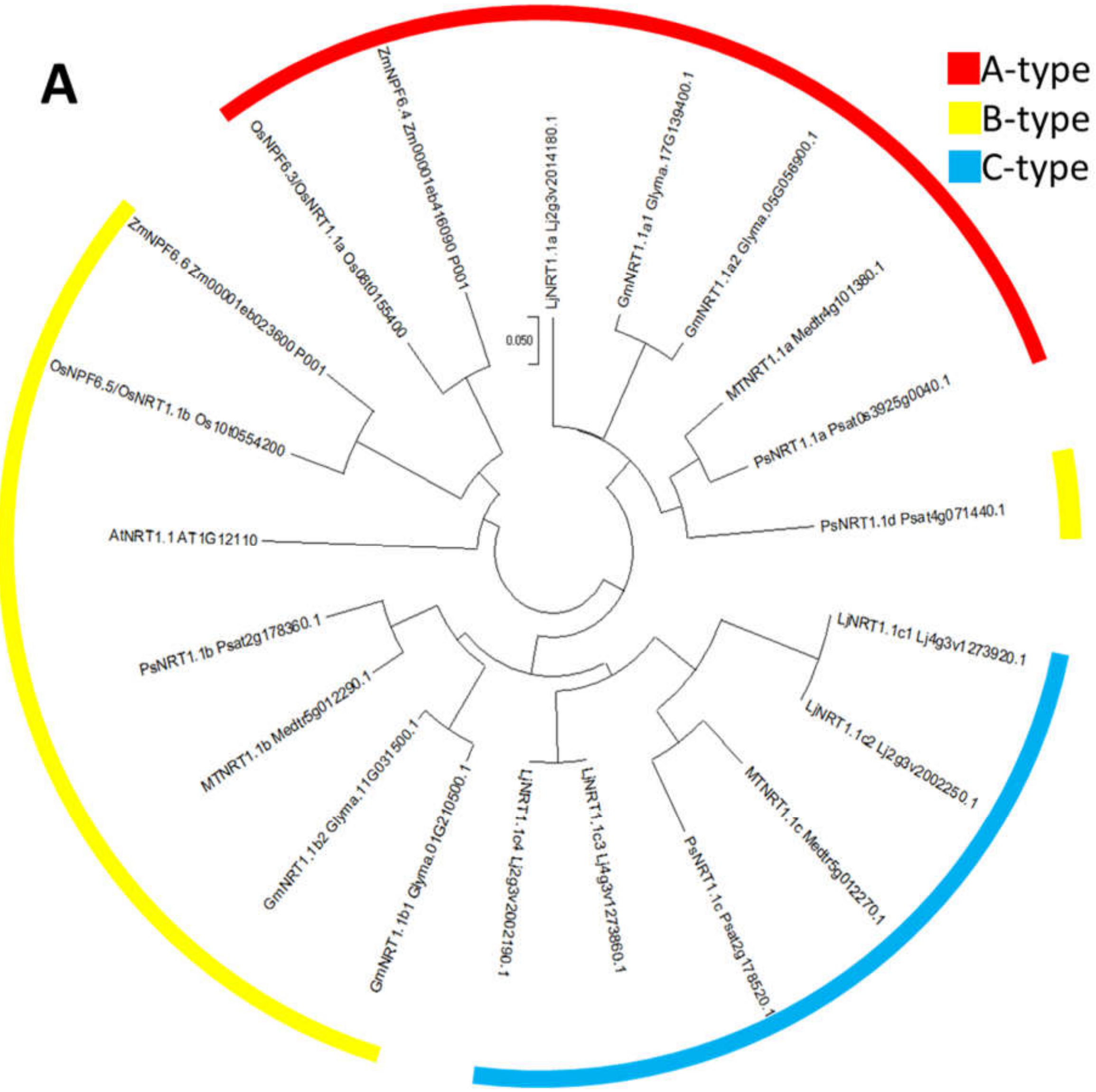

A
A-type
B-type
C-type
0.050
OsNPF6.3/OsNRT1.1a Os08t0155400
ZmNPF6.4 Zm00001eb416090 P001
LjNRT1.1a Lj2g3v2014180.1
GmNRT1.1a1 Glyma.17G139400.1
GmNRT1.1a2 Glyma.05G056900.1
MTNRT1.1a Medtr4g101380.1
PsNRT1.1a Psat0s3925g0040.1
PsNRT1.1d Psat4g071440.1
ZmNPF6.6 Zm00001eb023600 P001
OsNPF6.5/OsNRT1.1b Os10t0554200
AtNRT1.1 AT1G12110
PsNRT1.1b Psat2g178360.1
MTNRT1.1b Medtr5g012290.1
GmNRT1.1b2 Glyma.11G031500.1
GmNRT1.1b1 Glyma.01G210500.1
LjNRT1.1c4 Lj2g3v2002190.1
LjNRT1.1c3 Lj4g3v1273860.1
PsNRT1.1c Psat2g178520.1
MTNRT1.1c Medtr5g012270.1
LjNRT1.1c2 Lj2g3v2002250.1
LjNRT1.1c1 Lj4g3v1273920.1

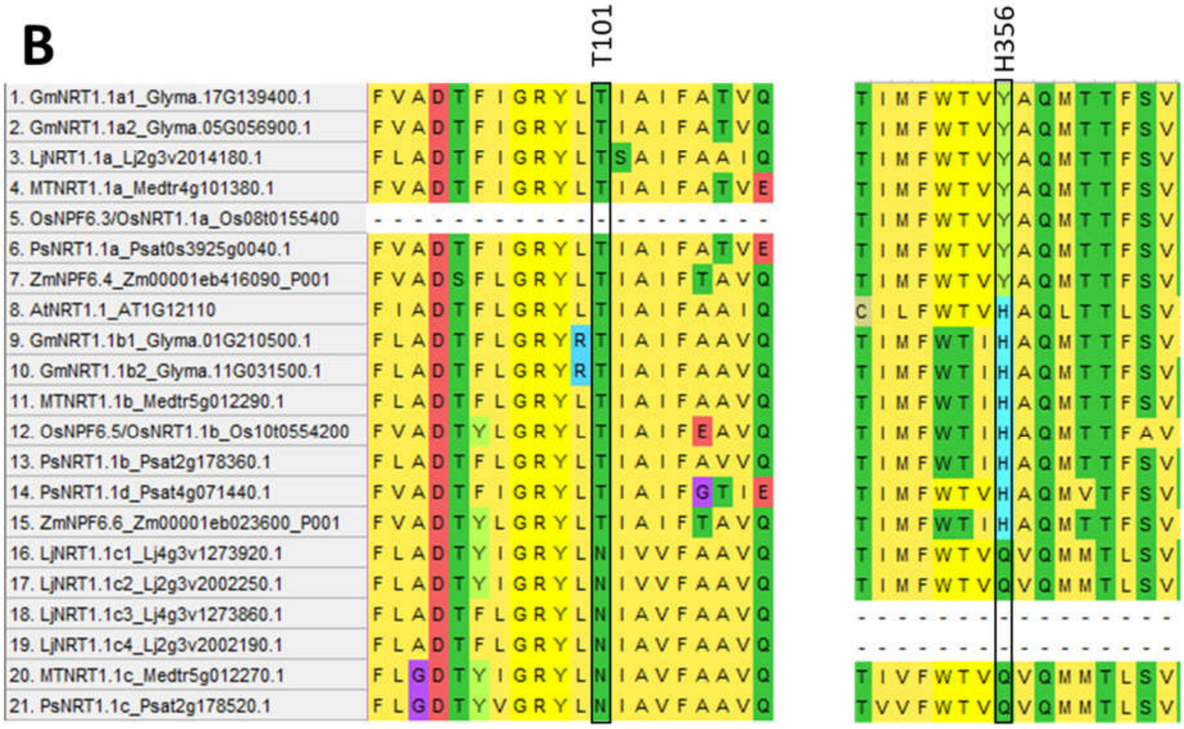

Supplemental Figure S2. Phylogenetic analysis of NITRATE TRANSPORTER 1.1 (NRT1.1) proteins (orthologues of AtNPF6.3) from *Arabidopsis thaliana, Medicago truncatula* and *Pisum sativum*

- **A.** Three NRT1.1 family subgroups. Red (subgroup-A), yellow (subgroup-B) and blue(subgroup-C). From the current Lotus japonicus genome, we found five NRT1.1s, which is one more than the reported by Criscuolo et al. (Plant Cell Environ. 2012 Sep;35(9):1567-81.doi: 10.1111/j.1365-3040.2012.02510.x.). A and B NRT1.1s from rice and maize clustered in the same clade, indicating parallel evolution of A and B subgroups in both monocot and dicot.
- **B.** The variation at amino acid (aa) Threonine (T) at aa 101 to Histidine (H) in C-subgroup and at amino acid Histidine at aa 356 to Tyrosine (Y) in A-subgroup and Glutamine (Q) in C-subgroup. The positions are corresponding to AtNRT1.1/AtNPF6.3.

We originally planned to include *Lotus japonicus* in the analysis of the NPF family, but to our great surprise, we found five LjNRT1.1 proteins, which is one more than the published paper (Plant Cell Environ. 2012 Sep;35(9):1567-81. doi: 10.1111/j.1365-3040.2012.02510.x. Epub 2012 Apr 19.). It looks as though the genome assembly and annotation of *Lotus japonicus* were renewed and new genes were found, so information about N transporters requires reviewing and we felt it was not a good model reference genome. For monocots, we found that both A and B subgroup NRT1.1 proteins from rice and maize clustered together as an outgroup of dicots proteins. This indicates that the nitrate sensing and signalling pathways are involved before the evolutionary separation of monocots and dicots. As this paper is to review transporters and signalling in pea, we only used the model plant of dicot, Arabidopsis, and model plant of legume, *Medicago truncatula*. Comparison between pea and rice will result variation between monocots and dicots, that is not specific for pea.

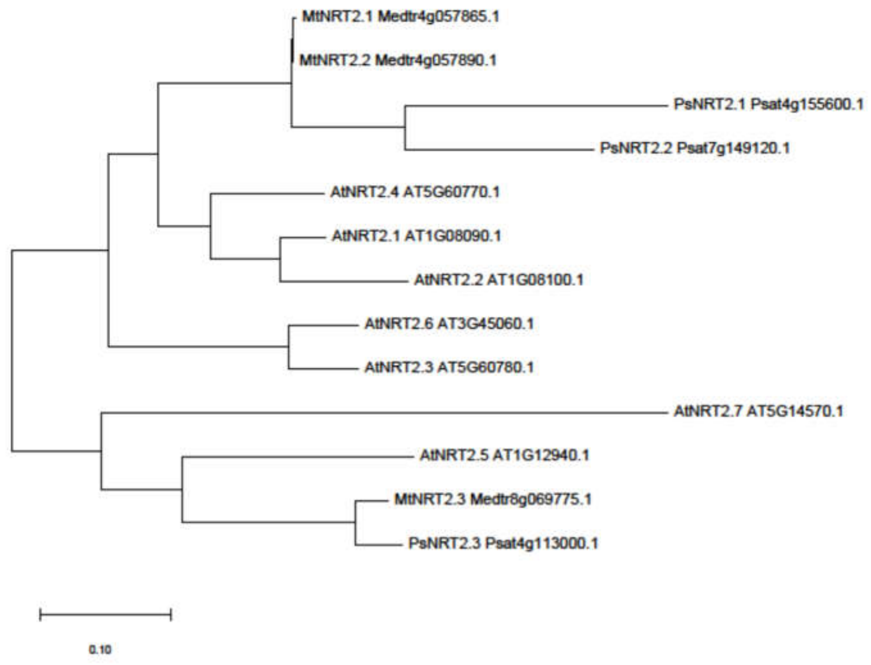

Supplemental Figure S3. A phylogenetic tree of NITRATE TRANSPORTER 2 (NRT2) from *Arabidopsis thaliana, Medicago truncatula* and *Pisum sativum***.**

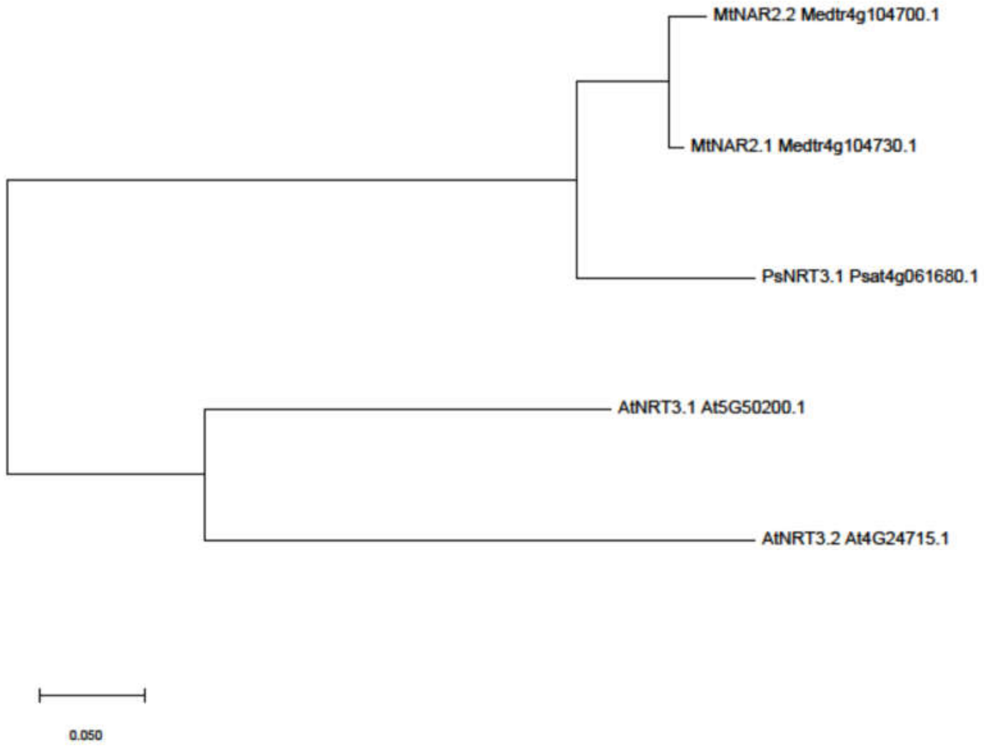

Supplemental Figure S4. A phylogenetic tree of High-affinity nitrate transporter-activating protein 2 (NAR2)/ NITRATE TRANSPORTER 3 (NRT3) proteins from *Arabidopsis thaliana, Medicago truncatula* and *Pisum sativum.*

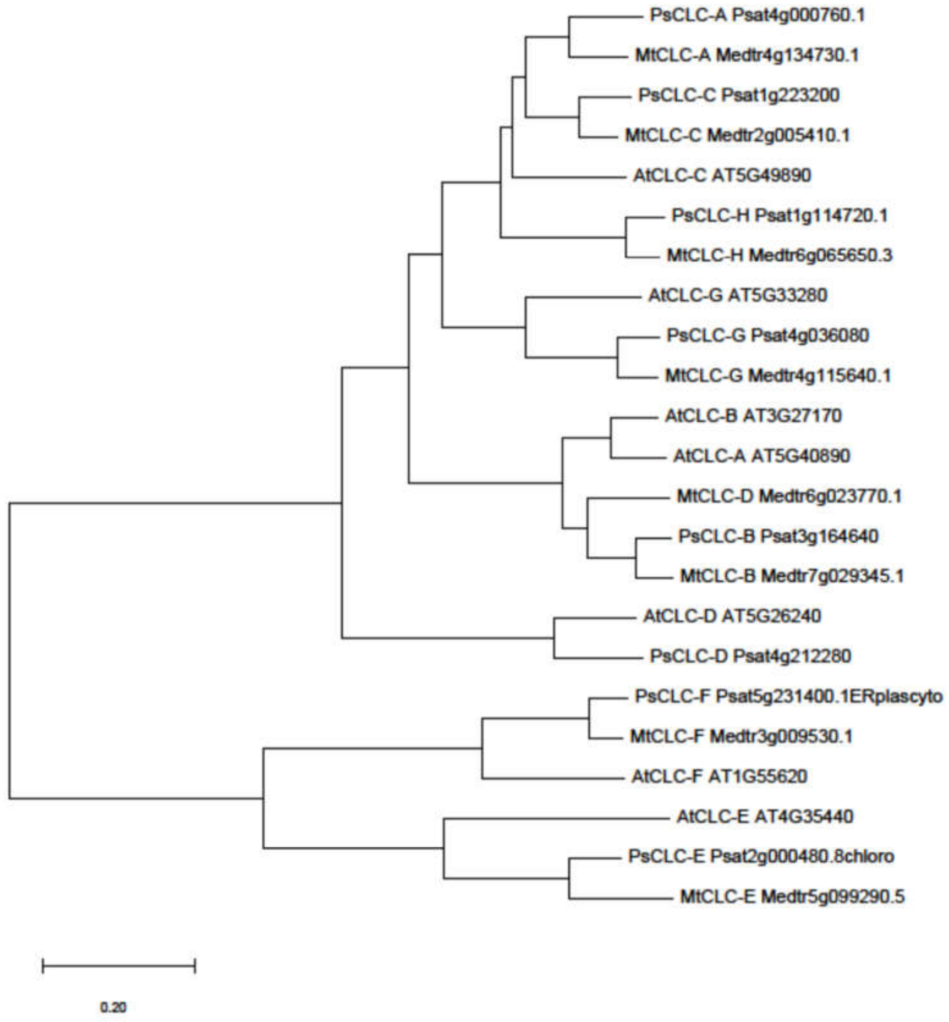

Supplemental Figure S5. A phylogenetic tree of CHLORIDE CHANNEL (CLC) proteins from *Arabidopsis thaliana, Medicago truncatula* and *Pisum sativum.*

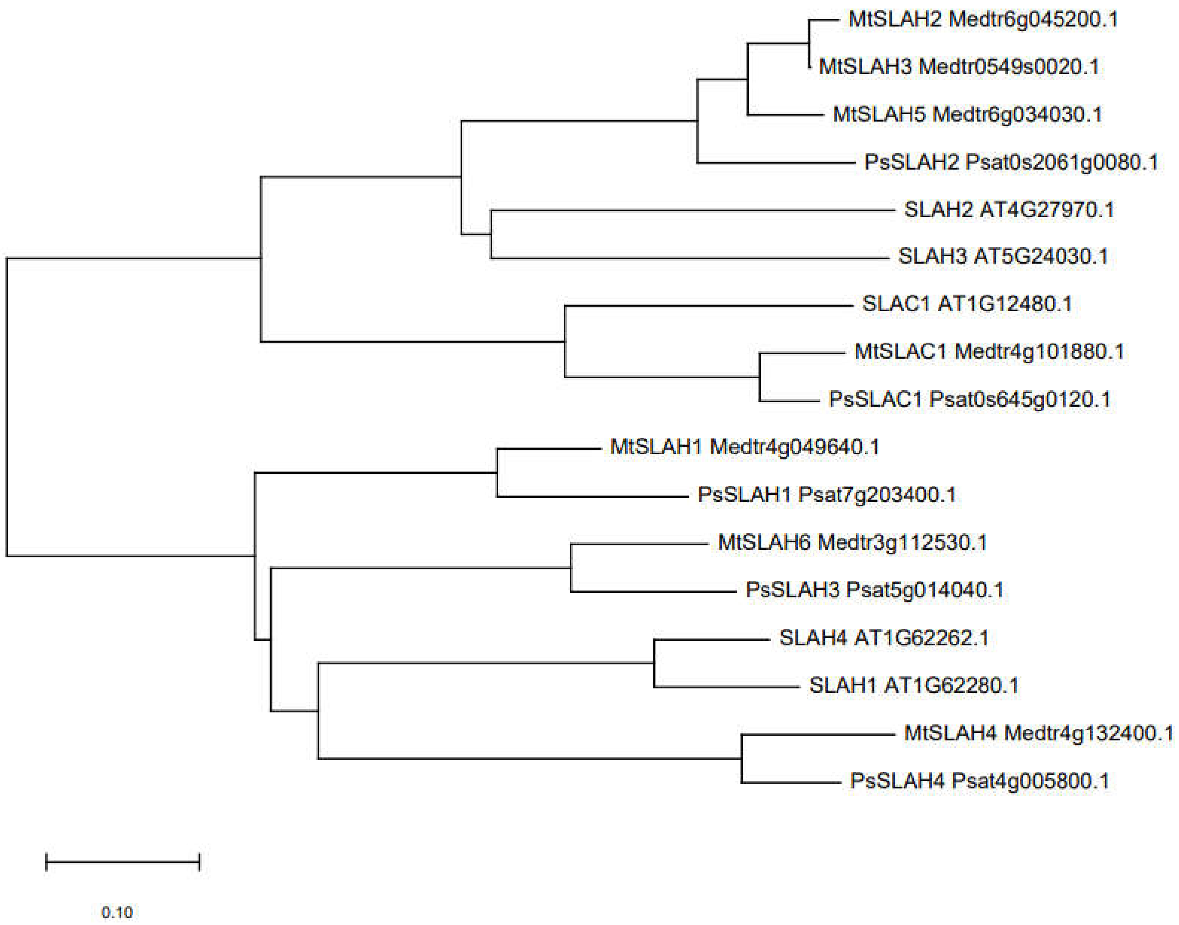

Supplemental Figure S6. A phylogenetic tree of SLOW ANION CHANNEL-ASSOCIATED 1 (SLAC1) and SLAC1 HOMOLOGUE (SLAH) family from *Arabidopsis thaliana, Medicago truncatula* and *Pisum sativum.*

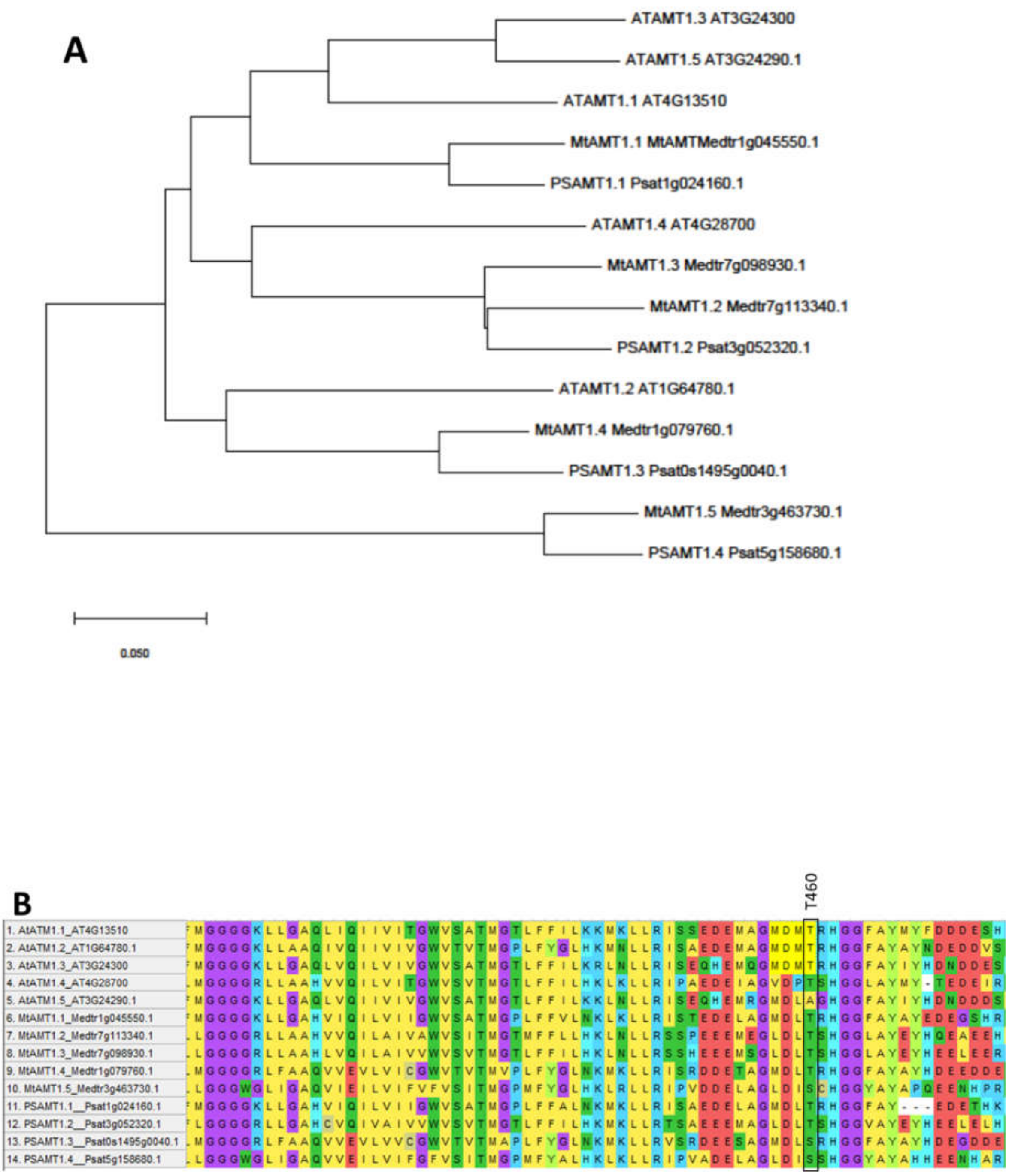
A
ATAMT1.3 AT3G24300
ATAMT1.5 AT3G24290.1
ATAMT1.1 AT4G13510
MtAMT1.1 MtAMTMedtr1g045550.1
PSAMT1.1 Psat1g024160.1
ATAMT1.4 AT4G28700
MtAMT1.3 Medtr7g098930.1
MtAMT1.2 Medtr7g113340.1
PSAMT1.2 Psat3g052320.1
ATAMT1.2 AT1G64780.1
MtAMT1.4 Medtr1g079760.1
PSAMT1.3 Psat0s1495g0040.1
MtAMT1.5 Medtr3g463730.1
PSAMT1.4 Psat5g158680.1
0.050
B
T460
1. AtATM1.1_AT4G13510
2. AtATM1.2_AT1G64780.1
3. AtATM1.3_AT3G24300
4. AtATM1.4_AT4G28700
5. AtATM1.5_AT3G24290.1
6. MtAMT1.1_Medtr1g045550.1
7. MtAMT1.2_Medtr7g113340.1
8. MtAMT1.3_Medtr7g098930.1
9. MtAMT1.4_Medtr1g079760.1
10. MtAMT1.5_Medtr3g463730.1
11. PSAMT1.1__Psat1g024160.1
12. PSAMT1.2__Psat3g052320.1
13. PSAMT1.3__Psat0s1495g0040.1
14. PSAMT1.4__Psat5g158680.1

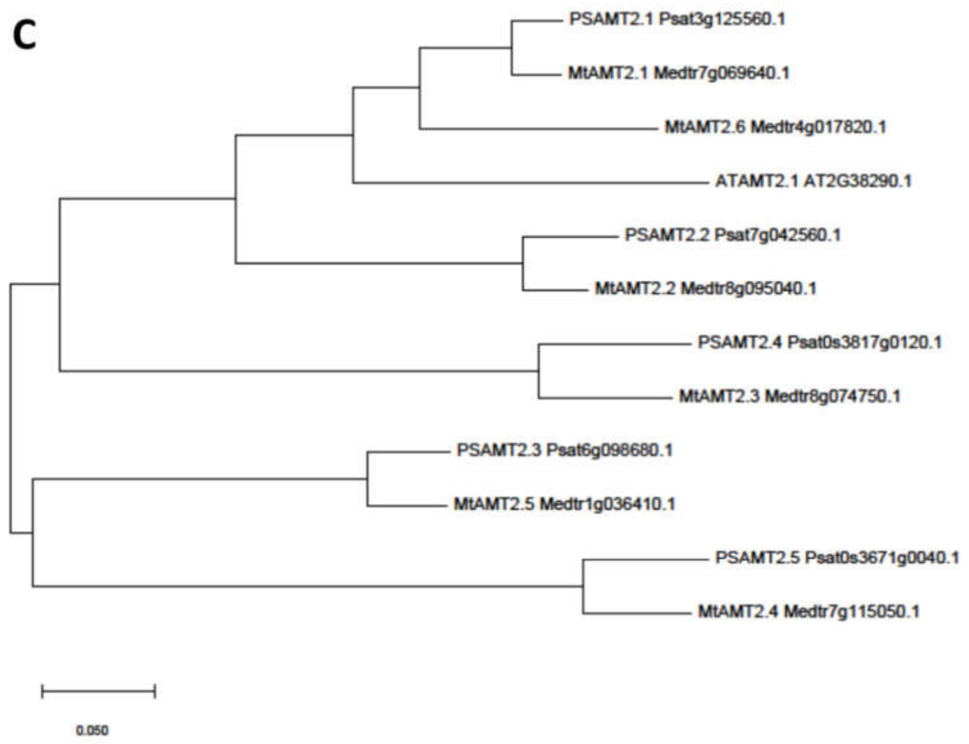

Supplemental Figure S7. A phylogenetic analysis of ammonium transporter (AMT) proteins from *Arabidopsis thaliana, Medicago truncatula* and *Pisum sativum.*

- **A.** A phylogenetic tree of AMT1s.
- **B.** Variations of phosphorylation site Threonine (T) 460 in AMT1.
- **C.** A phylogenetic tree of AMT2s.

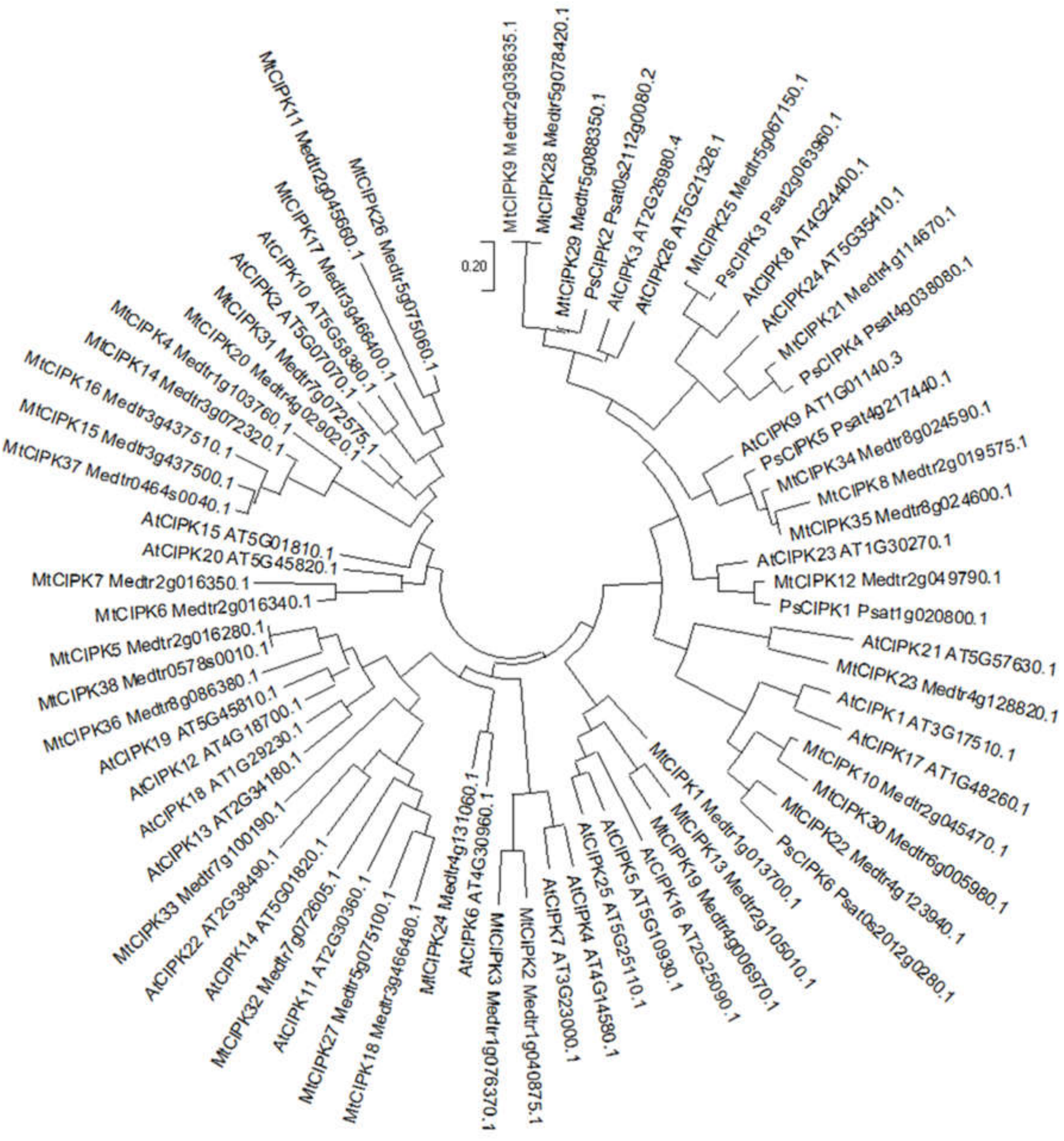

Supplemental Figure S8 A phylogenetic tree of CALCINEURIN B-LIKE PROTEIN INTERACTING PROTEIN KINASEs (CIPKs) from *Arabidopsis thaliana, Medicago truncatula* and *Pisum sativum.*

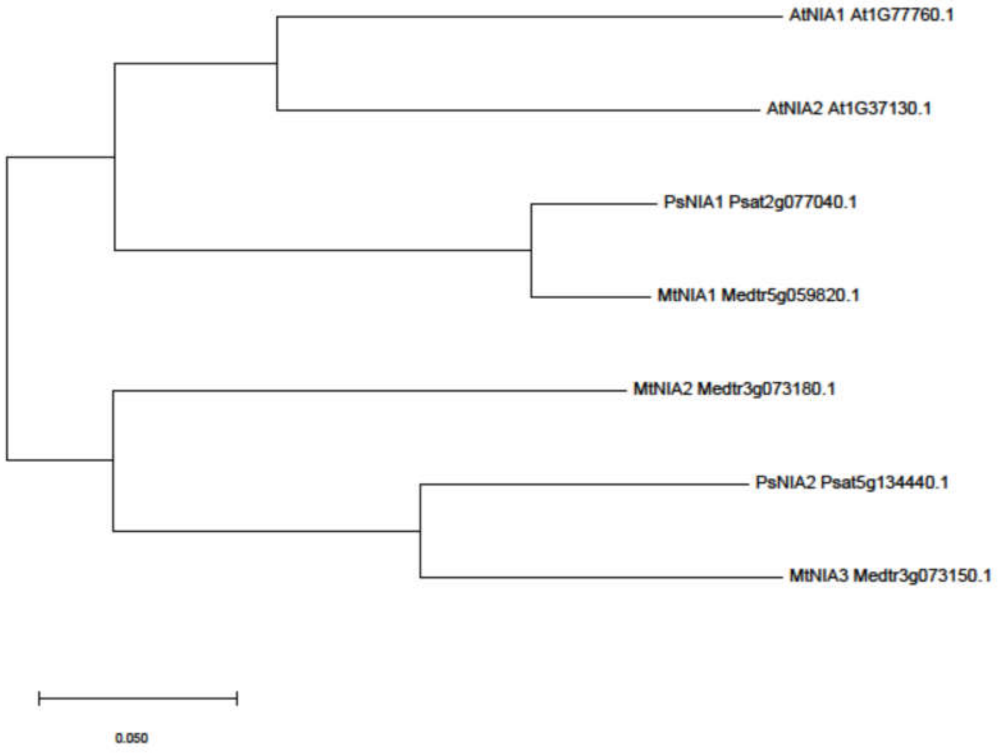

Supplemental Figure S9. A phylogenetic tree of NITRATE REDUCTASE (NIA) proteins from *Arabidopsis thaliana, Medicago truncatula* and *Pisum sativum.*

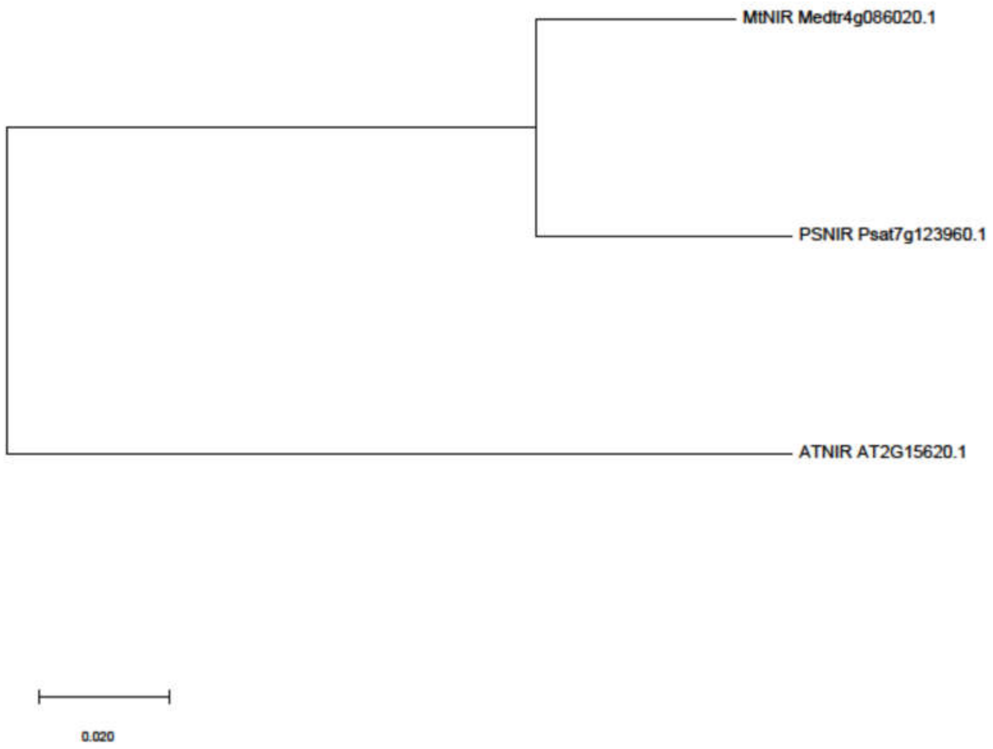

Supplemental Figure S10. A phylogenetic tree of NITRITE REDUCTASE (NIR) proteins from *Arabidopsis thaliana, Medicago truncatula* and *Pisum sativum.*

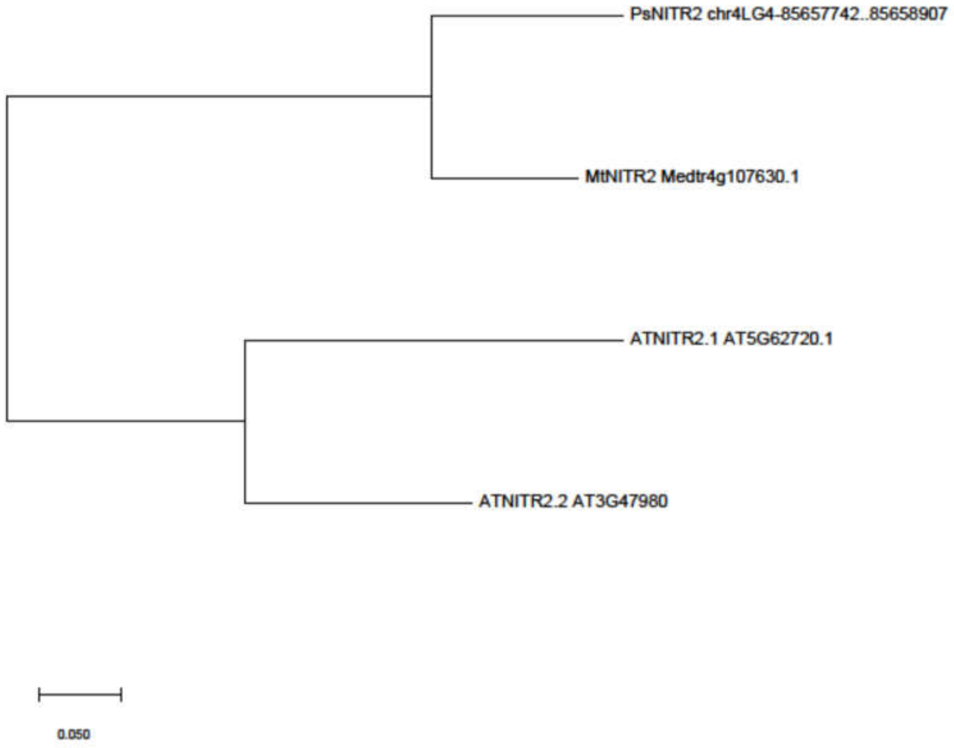

Supplemental Figure S11. A phylogenetic tree of NITR2 proteins from *Arabidopsis thaliana, Medicago truncatula* and *Pisum sativum.*

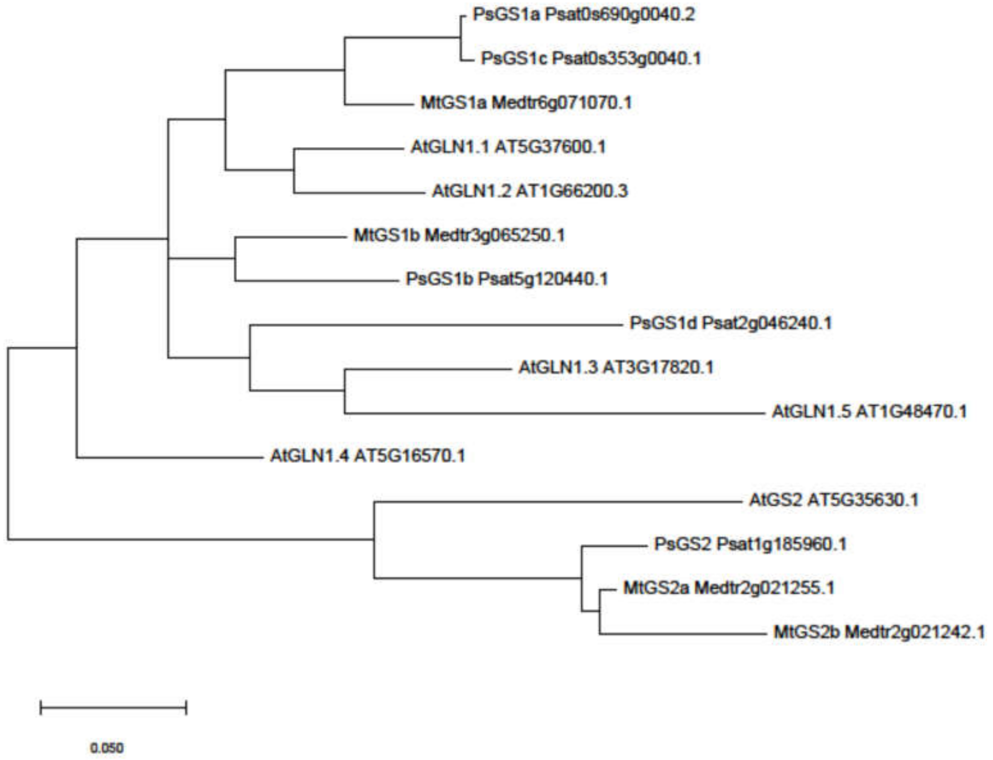

Supplemental Figure S12. A phylogenetic tree of glutamine synthetases (GS) in *Arabidopsis thaliana, Medicago truncatula* and *Pisum sativum*

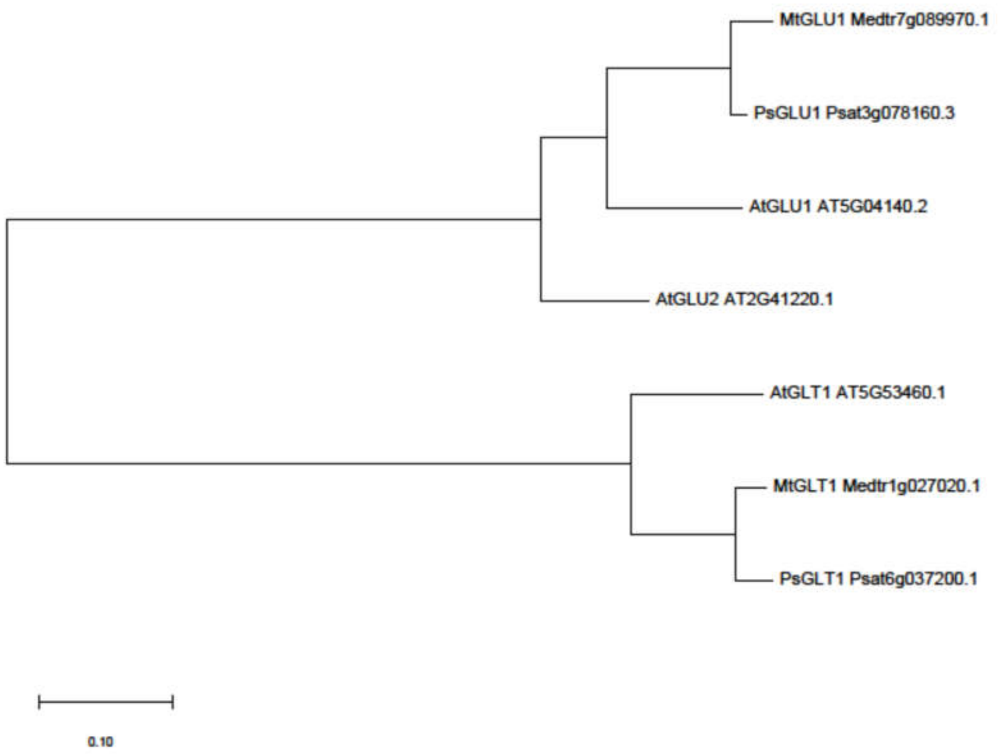

Supplemental Figure S13. A phylogenetic tree of GLUTAMATE SYNTHASE (GLU) and GLUCOSE TRANSPORTER(GLT) from *Arabidopsis thaliana, Medicago truncatula* and *Pisum sativum.*

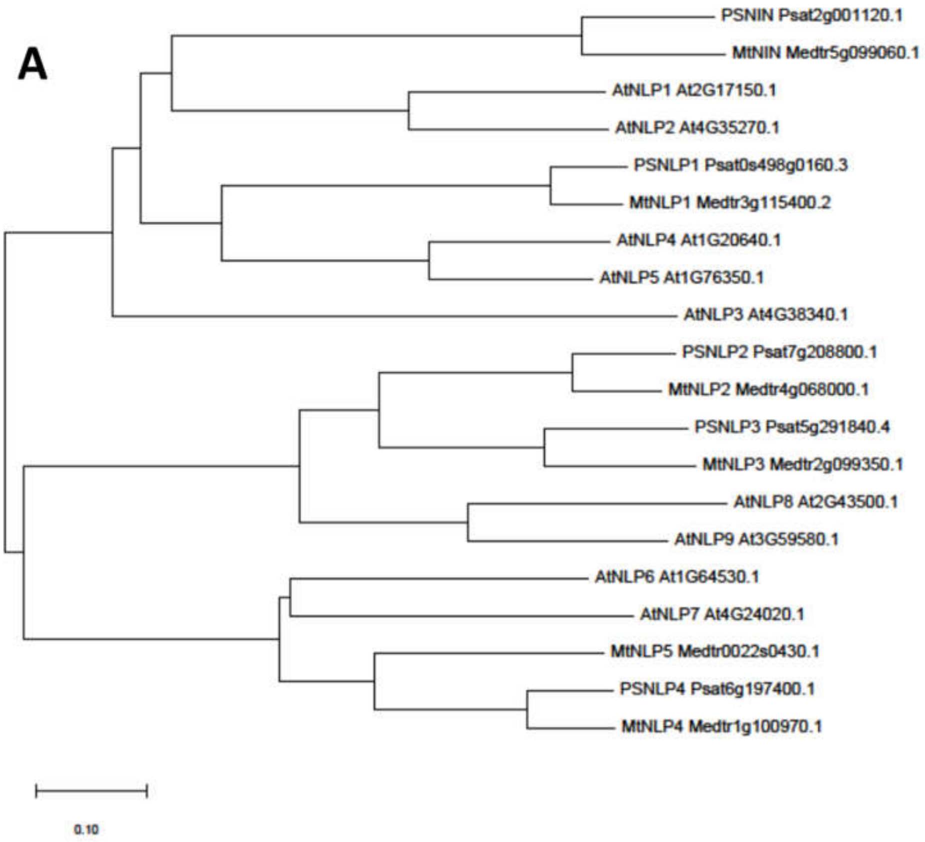

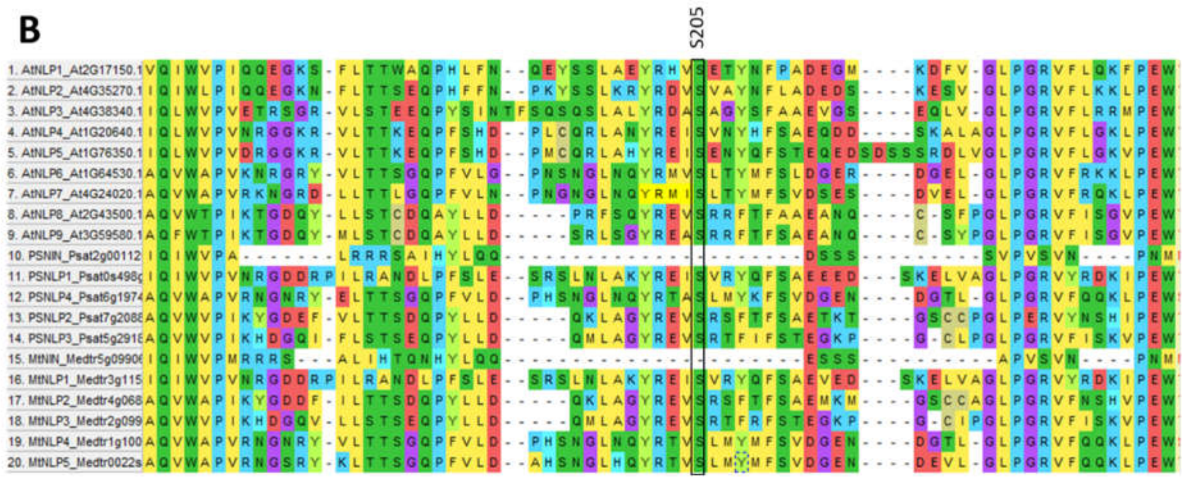

Supplemental Figure S14. A phylogenetic analysis of NODULE INCEPTION (NIN)-LIKE PROTEIN (NLP) proteins from *Arabidopsis thaliana, Medicago truncatula* and *Pisum sativum.*

- **A.** A phylogenetic tree of NLP proteins.
- **B.** Variations at the phosphorylated site Serine (S) 205. Amino acid position is according to AtNLP7.

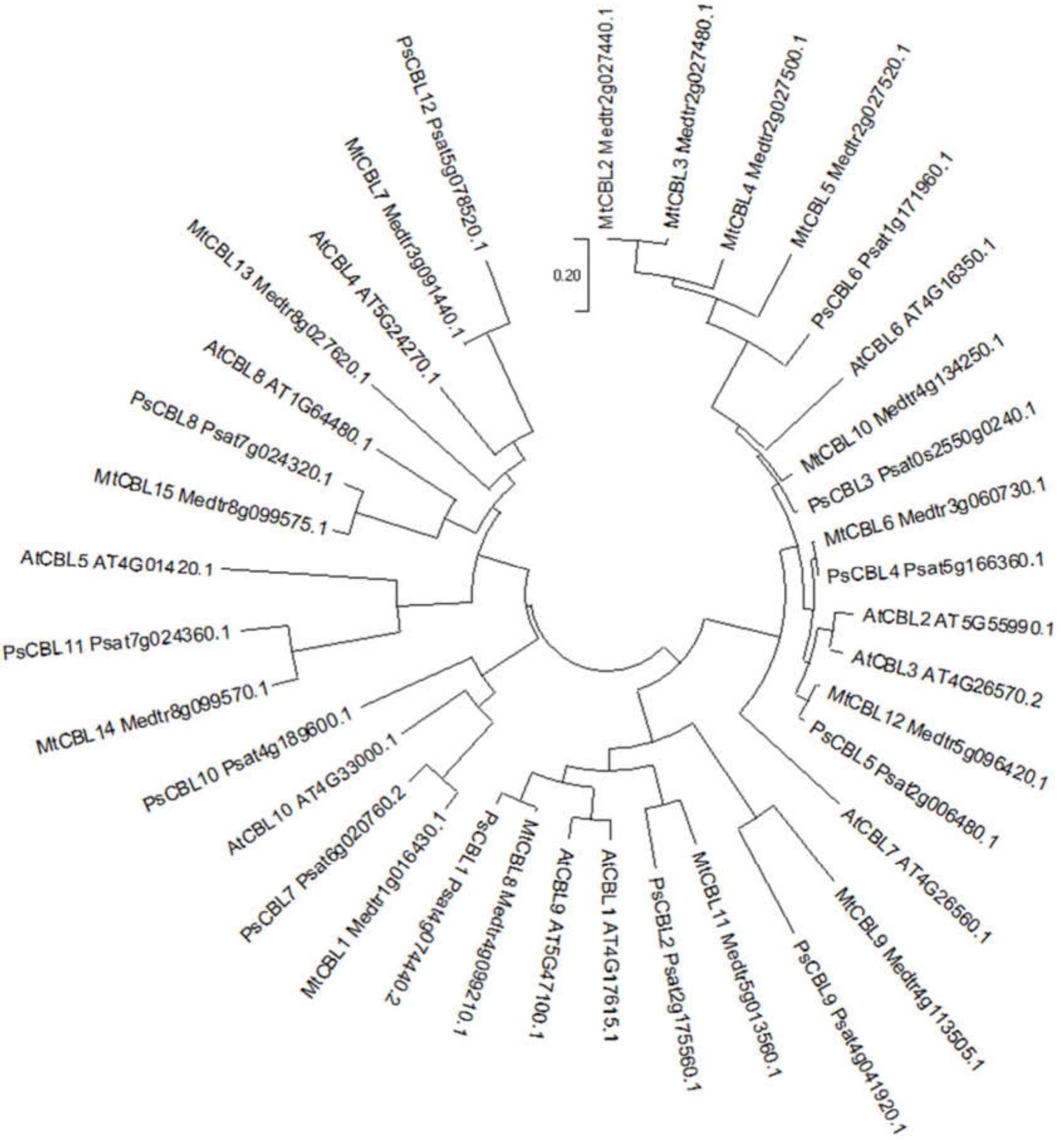

Supplemental Figure S15. A phylogenetic tree of CALCINEURIN B-LIKE (CBL) proteins from *Arabidopsis thaliana, Medicago truncatula* and *Pisum sativum.*

c.

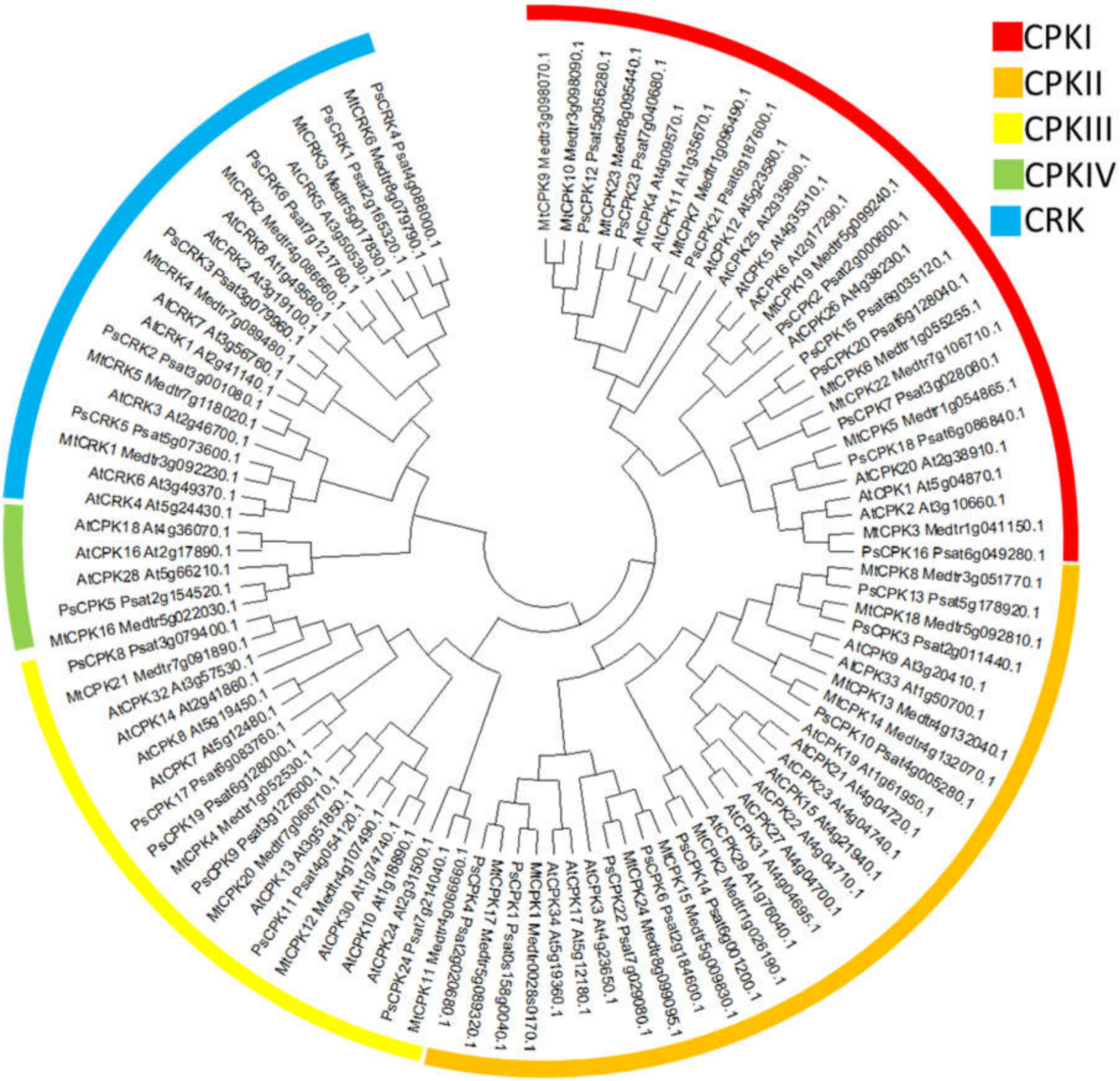

Supplemental Figure S16. A phylogenetic tree of CALCIUM-DEPENDENT PROTEIN KINASE (CPK) and CALCIUM-DEPENDENT PROTEIN RELATED KINASE (CRK) proteins from *Arabidopsis thaliana, Medicago truncatula* and *Pisum sativum.*

Four subgroups of CPK and CRK group are indicated by red (CPKI), orange (CPKII), yellow (CPKIII), green (CPKIV) and blue (CRK).

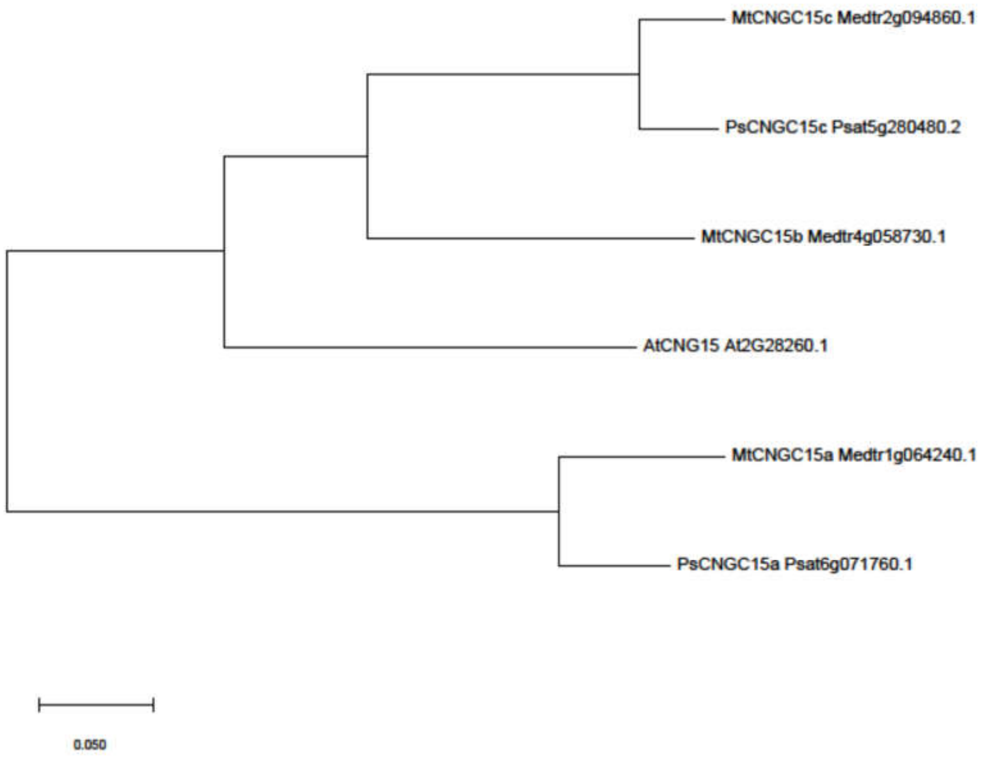

Supplemental Figure S17. A phylogenetic tree of CYCLIC NUCLEOTIDE-GATED CHANNEL 15 (CNGC15) proteins from *Arabidopsis thaliana, Medicago truncatula* and *Pisum sativum.*

A

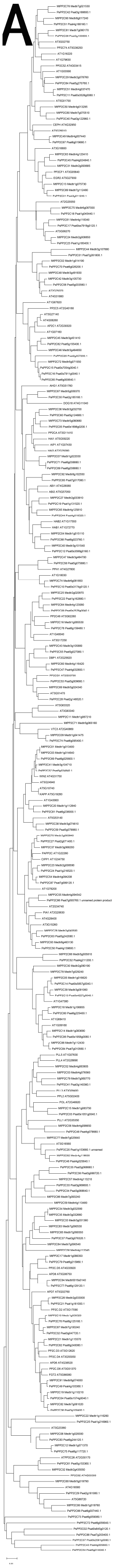

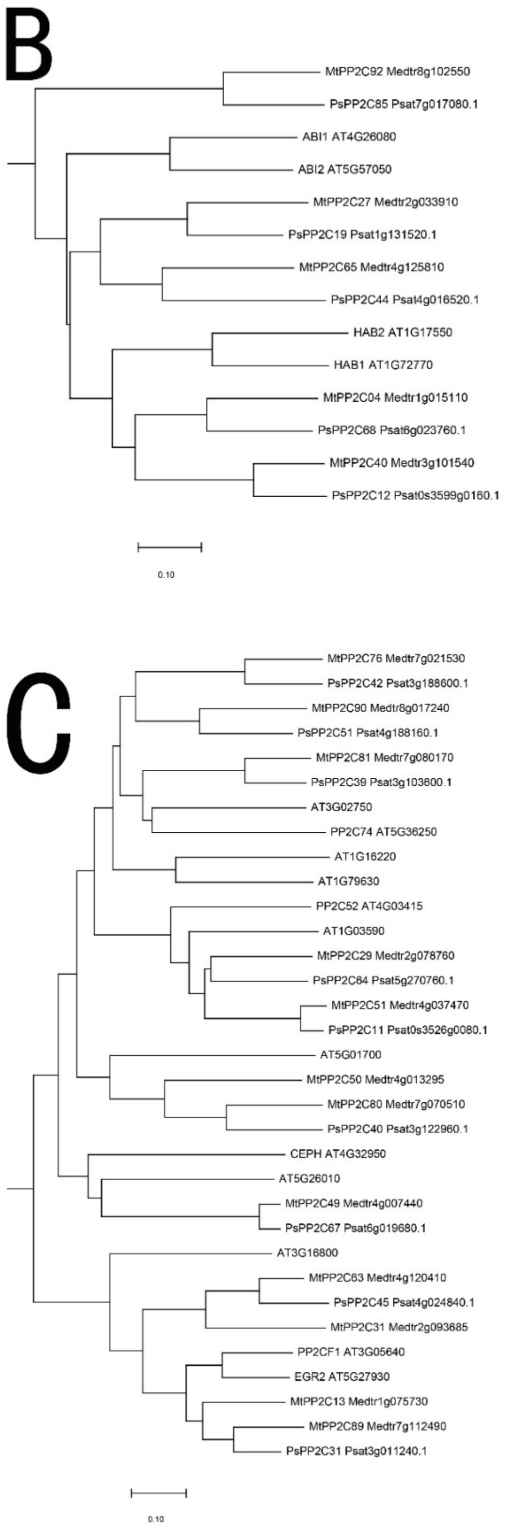

Supplemental Figure S18. A phylogenetic tree of Protein phosphatase 2C (PP2C) proteins from *Arabidopsis thaliana, Medicago truncatula* and *Pisum sativum.*

A. The whole phylogenetic tree.
B. Branch of ABA INSENSITIVE 1 (ABI1).
C. Branch of CEPD-INDUCED PHOSPHATASE (CEPH).

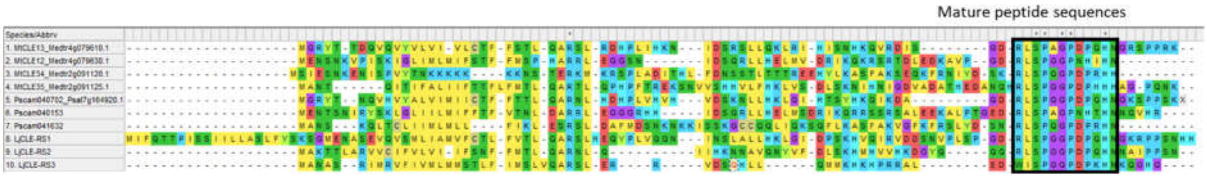

Supplemental Figure S19. An alignment of reported CLAVATA3/Embryo Surrounding Region-Related (CLE) peptides induced by nitrate and/or rhizobia. Black rectangle indicates sequences of mature peptides.

Supplemental Table S1. Data and condition of nitrate content in Figure 2

Pea Non-inoculation		μmol/g FW	Nitrate condition	Reference
	1	13.85	2mM Nitrate	27
	2	12.2	2mM Nitrate	26
Pea Inoculated				
	1	9.19	2mM Nitrate	27
	2	13.75	2mM Nitrate	26
	3	5	4mM Nitrate	25
Arabidopsis				
	1	129.15	0.2mM Nitrate	16
	2	147.22	6mM Nitrate	28
	3	94.25	0.2mM Nitrate	29
Wheat				
	1	77	5mM Nitrate	32
	2	59.8	Nitrate applied	30
	3	29.64	Nitrate applied	31

FW: Fresh weight
